# Curcumin: An Anti-Inflammatory Molecule from a Curry Spice on the Path to Cancer Treatment

**DOI:** 10.3390/molecules16064567

**Published:** 2011-06-03

**Authors:** Purusotam Basnet, Natasa Skalko-Basnet

**Affiliations:** Drug Transport and Delivery Research Group, Department of Pharmacy, University of Tromsø, Tromsø N-9037, Norway; Email: natasa.skalko-basnet@uit.no (N.S.-B.)

**Keywords:** curcumin, cancer, anti-oxidant, anti-inflammatory, nanoparticles

## Abstract

Oxidative damage and inflammation have been pointed out in preclinical studies as the root cause of cancer and other chronic diseases such as diabetes, hypertension, Alzheimer’s disease, *etc*. Epidemiological and clinical studies have suggested that cancer could be prevented or significantly reduced by treatment with anti-oxidant and anti-inflammatory drugs, therefore, curcumin, a principal component of turmeric (a curry spice) showing strong anti-oxidant and anti-inflammatory activities, might be a potential candidate for the prevention and/or treatment of cancer and other chronic diseases. However, curcumin, a highly pleiotropic molecule with an excellent safety profile targeting multiple diseases with strong evidence on the molecular level, could not achieve its optimum therapeutic outcome in past clinical trials, largely due to its low solubility and poor bioavailability. Curcumin can be developed as a therapeutic drug through improvement in formulation properties or delivery systems, enabling its enhanced absorption and cellular uptake. This review mainly focuses on the anti-inflammatory potential of curcumin and recent developments in dosage form and nanoparticulate delivery systems with the possibilities of therapeutic application of curcumin for the prevention and/or treatment of cancer.

## 1. Background

Polyphenols play an important role in the maintenance of health and prevention of diseases. Polyphenols in the human diet are derived mainly from vegetables, fruits and spices. Drinks and beverages such as coffee, green and black tea, as well as chocolate, red wine, olive oil, nuts, *etc*., are also good sources of polyphenols. Many of these polyphenol-rich natural resources have been traditionally used as medicines for the prevention of diseases, as well as maintenance of youth and longevity. The recent line of studies has confirmed that these traditionally used natural remedies are strong anti-oxidant and anti-inflammatory agents. In addition, many of them play important roles in regulating the immune system and are now being investigated as chemopreventive, neuroprotective, cardioprotective and hepatoprotective agents, either acting alone or in combinations [1,2]. In particular, turmeric, a typical example of polyphenol-rich natural remedies, has been used for centuries in Indian traditional medicine (Ayurveda) and Traditional Chinese Medicine (TCM) [2]. Moreover turmeric, a popular curry spice, is also used as a food additive and preservative agent worldwide. Turmeric products have been characterized as safe by the Food and Drug Administration (FDA) in the USA, the Natural Health Products Directorate of Canada, and the Joint Expert Committee of the Food and Agriculture Organization/World Health Organization (FAO/WHO) [3]. Curcumin, a principal polyphenol component of turmeric, is available as an over-the-counter (OTC) supplement worldwide. Turmeric was introduced to Europe in the 13th century by Marco Polo and perhaps surprisingly, apart from an early study published in *The Lancet* in 1937, curcumin/turmeric has only entered extensive preclinical studies and scientific phase I and II/III clinical trial levels in the last 10–15 years [2,4].

Current literature (according to the SciFinder database, March 2011) showed a total of 12,032 hits on “curcumin”. Among them, 54 references were of clinical trials, 1,016 references in a form of review and 1,408 references on patent applications. In the same database, the numbers of references during 1990 to 2000 were 1463, 7, 106, and 118 for total, clinical trials, reviews, and patent applications, respectively, and there were only a total number of 600 references on curcumin recorded from 1950 to 1989. At the time of writing this review, a total of 56 clinical trials (phase I and phase II) on curcumin are listed on the website of the USA National Institutes of Health [5]. Among them, 16 trials were already completed, three were terminated, one was withdrawn and the rest of the studies are on-going. Such a vast number of researches on curcumin mainly targeting its therapeutic applications hold the promise of an interesting outcome in the near future.

Our particular interest are the anti-oxidative and anti-inflammatory properties of curcumin, which might provide a therapeutic window for the treatment of cancer. Curcumin downregulates various pro-inflammatory cytokine expressions such as tumor necrosis factor (TNF-α), interleukins (IL-1, IL-2, IL-6, IL-8, IL-12) and chemokines, most likely through inactivation of the nuclear transcription factor, nuclear factor (NF)-κB. Likewise, curcumin is known to decrease the inflammation associated with experimental colitis, including a substantial reduction of the rise in myleoperoxidase (MPO) activity, an established marker for inflammatory cells (mainly polymorphonuclear leukocytes) and TNF-*α* [6,7]. In addition, curcumin is able to reduce colonic nitrite levels and downregulate cyclooxygenase (COX)-2, inducible nitric oxide synthase (*i*NOS) expression and p38 mitogen activated protein kinase (MAPK) activation [6,7]. In spite of the abundant evidence at the molecular level, and extensive studies at the preclinical and clinical levels, its therapeutic outcome remains a challenge owing to its low solubility and poor bioavailability. Therefore, this review focuses particularly on the recent developments in dosage forms and nanoparticulate delivery systems for curcumin designed to enhance the absorption, cellular uptake, bioavailability and ultimate efficiency.

## 2. Turmeric History

Turmeric is prepared by grinding of dried rhizomes mainly from *Curcuma longa* L. of the Zingiberaceae family. These powdered dried rhizomes have at least 76 synonyms listed in the 1999 WHO monograph [8]. Among them some important names are *Haldi* (in Hindi), *Haridra* or *Gauri* (in Sanskrit), *Chiang Huang* (in Chinese), *Ukon* (in Japanese), *Kurkum* (in Arabic), *Besar* (in Nepali), *etc*.

**Figure 1:**
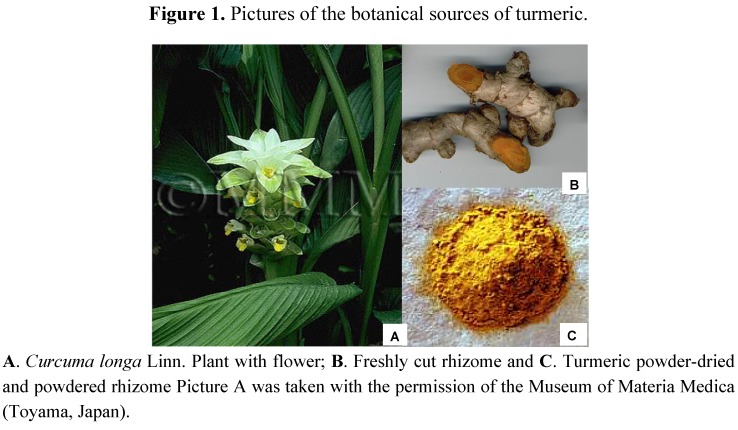
Pictures of the botanical sources of turmeric.

*Curcuma* *longa* grows naturally throughout the Indian subcontinent and in tropical climates. It is a short-stemmed perennial, which grows to up to 100 cm in height. It has curved, oblong, and ovate leaves with beautiful white to colourful flower and cylindrical rhizomes (Figure 1). India produces most of the World’s supply of turmeric [9,10]. More recently, it has been used by the food industry as additive, flavouring, preservative, and colouring agent (e.g., in mustard, margarine, soft drinks, and beverages). As a safe colouring agent, curcumin is listed in the international numbering system for food additives with the code E100. Non-medical applications of turmeric include cosmetics, particularly in Hindu rituals and ceremonies. Commercially available turmeric may contain essential oils, polyphenols, protein, fat, minerals, carbohydrates, and moisture [11]. The aromatic properties of turmeric are thought to be attributable to its volatile essential oils [12].

As a traditional medicine, turmeric has also been extensively used for centuries to treat a diversity of disorders including rheumatism, body aches, skin diseases, wounds, intestinal worms, diarrhoea, intermittent fevers, hepatic disorders, biliousness, urinary discharges, dyspepsia, inflammation, constipation, leukoderma, amenorrhoea and colic inflammation [2]. Besides the traditional literature, the first recorded scientific article referring to *Curcuma* spp. was published in 1748, and the first pharmacologic review of turmeric appeared 67 years later [3,13].

## 3. Curcumin Chemistry

**Figure 2:**
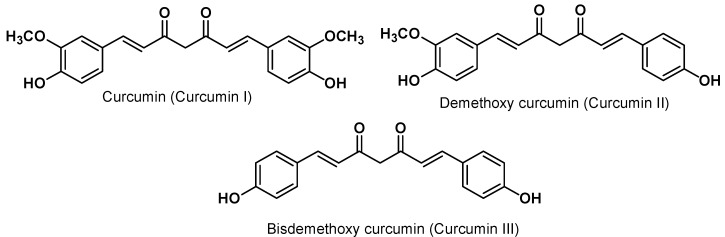
Structure of three major curcuminoids in turmeric.

The yellow colour of turmeric is mainly due to the presence of polyphenolic curcuminoids, which constitute approximately 3% to 5% of most turmeric preparations. The alcoholic extract of turmeric mainly contains three curcuminoids, namely curcumin (also referred as curcumin I or diferuloylmethane), desmethoxycurcumin (curcumin II), and bisdesmethoxycurcumin (curcumin III) (Figure 2). The first attempt at curcumin purification was carried out by Vogel and Pelletier in 1815 and its structure as diferuloylmethane was established in 1910 [13,14]. Its chemical structure was confirmed in 1973 by Roughley and Whiting [15] and the solution structure was only confirmed in 2007 by Payton *et al.* [16].

It should be noted that most commercially available “curcumin” is not a pure curcumin, but rather is a mixture of curcumin (approx. 77%), desmethoxycurcumin (approx. 18%) and bisdesmethoxy- curcumin (approx. 5%) [17]. Curcuminoids are also reported from *C. aromatica*, *C. phaecaulis*, *C. zedoaria*, *C. xanthorrhiza*, *C. mangga* among the more than 120 *Curcuma* plants identified so far. Among the curcuminoids, curcumin is the main and biologically targeted phytochemical and *C. longa* is the main source of curcumin [2].

**Scheme 1:**
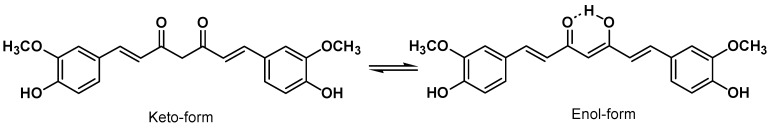
pH dependent keto- and enol- tautomeric form of curcumin.

Curcumin [chemical name: (*E*,*E*)-1,7-bis(4-hydroxy-3-methoxyphenyl)-1,6-heptadiene-3,5 dione) is a bis-α,β-unsaturated β-diketone. It has a molecular weight of 368.38, a melting point of 179–183 °C, and chemical formula of C_21_H_20_O_6_. Under physiological conditions, curcumin can exist in both an enol and a bis-keto form, which coexist in equilibrium (Scheme 1).

Curcumin is readily soluble in dimethylsulfoxide (DMSO), ethanol or acetone, but it is sparingly soluble in water. In acidic and neutral solutions as well as in the solid state, the keto form predominates, and curcumin acts as a potent donor of H-atoms. In contrast, under alkaline conditions (≥pH 8), the enolic form predominates, and the phenolic part of the molecule plays the principal role as an electron donor [18]. In solution, it has been demonstrated that 90% of curcumin was found degraded to *trans*-6-(4’-hydroxy-3’-methoxyphenyl)-2,4-dioxo-5-hexanal, vanillin, feruloylmethane, and ferulic acid within 30 minutes [19]. Curcumin is similarly unstable at basic pH, but in presence of calf serum or human blood less than 20% of curcumin was found to be decomposed in 1 h [20]. Addition of antioxidants (ascorbic acid, *N-*acetylcysteine or glutathione) to culture media also inhibited the degradation of curcumin. Yellow curcumin changes to dark red colour at alkaline pH and under physiological conditions the *λ*_max_ for curcumin is observed at 420 nm [21].

## 4. Curcumin Safety and Toxicity

Small doses of turmeric (curcumin) are taken daily as a spice by the population in many Asian countries. In one epidemiologic survey, in terms of its dietary use in Nepal, turmeric consumption was found to be up to 1,500 mg per person per day, equivalent to approx. 50 mg/day of curcumin [22]. In India, where the average intake of turmeric can be as high as 2,000–2,500 mg per day (corresponding to approx. up to 100 mg of curcumin), no toxicities or adverse effects have been reported or studied at the population level [23]. However the doses administered in clinical trials are expected to be rather higher than those normally consumed in the diet. This fact underlines the need for systematic safety and toxicity studies. Based on repeated studies, turmeric is Generally Recognized As Safe (GRAS) by the US FDA, and curcumin has been granted an acceptable daily intake level of 0.1–3 mg/kg-BW by the Joint FAO/WHO Expert Committee on Food Additives, 1996 [3].

In the systematic studies funded by the Prevention Division of the US National Cancer Institute (NCI) conducted in rats, dogs, or monkeys and at oral doses of curcumin up to 3,500 mg/kg-BW for up to 90 days, no adverse effects were observed [3]. In a preclinical study involving the administration of 2% dietary curcumin (approx. 1,200 mg/kg-BW) to rats for 14 days [24] or in a study of 0.2% dietary curcumin (approx. 300 mg/kg-BW) administered to mice for 14 weeks, no toxicity was observed [25]. Furthermore, a reproductive toxicity study with the oral curcumin administration of up to 1,000 mg/kg-BW daily, no toxicity was observed in two successive generations in rats [26].

In a study performed in India, daily administration of 1,200–2,100 mg of oral curcumin to patients with rheumatoid arthritis for 2–6 weeks did not cause any toxicity [27]. In another study of high-dose oral curcumin by Cheng *et al.* in Taiwan, administration of 500, 1,000, 2,000, 4,000, and 8,000 mg of curcumin daily for three months to patients with preinvasive malignant or high risk premalignant conditions, no noticeable adverse effects were detected [28]. Lao *et al.* studied safety of curcumin in healthy volunteers using curcumin capsules (containing 75% curcumin, 23% demethoxycurcumin and 2% bisdemethoxycurcumin) with single escalating doses from 500 mg to 12,000 mg. Among 24 enrolled healthy subjects, seven developed adverse effects, including diarrhea, headaches, rashes and yellowish stools. All toxicities observed were of grade 1 and not related to the doses. In this study, the maximum tolerated dose could not be determined because more than 12,000 mg of curcumin is regarded as too bulky [29].

In a phase I clinical trial on oral curcumin in patients with advanced colorectal cancer in which the US NCI criteria were used to assess potential toxicity, curcumin was well tolerated at all dose levels up to 3,600 mg daily for up to four months [30]. Adverse effects related to curcumin consumption reported by patients in these studies were mainly gastrointestinal (nausea and diarrhoea). Two abnormalities were detected in blood tests in this trial, both possibly related to treatment: an increase in serum alkaline phosphatase level was observed in four patients (two NCI grade 1, and two grade 2); and three other patients had serum lactate dehydrogenase increases to >1.5 times the upper limit of normal. It is not clear whether these abnormal blood test results were related to the activity of the malignant disease in these patients or to treatment toxicity.

Although turmeric is often used to treat inflammatory skin conditions in traditional Asian medical systems, it should be noted by potential laboratory and clinical investigators that a few reports of allergic dermatitis after contact with curcumin have been published in the scientific literature [31,32,33]. An allergic reaction to turmeric-related products was also described in one healthy volunteer enrolled in a phase I study testing the safety of turmeric oil and turmeric extract [34]. Despite the lack of systematic testing on the interaction between curcumin with other commonly used drugs, the US Department of Health and Human Services has recommended, based on published laboratory and animal studies, that co-administration of curcumin with nonsteroidal anti-inflammatory drugs (NSAIDs) or anti-coagulant drugs (heparin, clopidogrel, aspirin) may result in an increased risk of bleeding. They have also suggested that interference may be found with other drugs that affect or are metabolized by the cytochrome P450 (CYP) enzyme system, resulting in the potential for erratic drug levels in blood [7]. In addition to this advice, it has been speculated that *Curcuma* extract (rather than curcumin) may potentially interfere with histamine 2-receptors antagonists (e.g., ranitidine) and proton-pump inhibitors (e.g., omeprazole) *via* inhibitory effects on histamine receptors [35]. Based on animal studies, other scientists have proposed that curcumin may enhance the hypoglycaemic effect of anti-diabetic medication or the efficacy of anti-lipemic drugs, *via* inhibition of the CYP enzyme system or reducing the low-density lipoprotein fraction in the blood [36,37].

Curcumin is an approved food colouring agent in addition to a dietary item. In spite of reported minor adverse effects, large doses of up to 12,000 mg per day of curcumin were found to be well tolerated in humans. Therefore, based on the safety and toxicity profile, in several clinical trials the targeted doses for curcumin can be recommended in between 4,000–8,000 mg to obtain the maximum therapeutic effects.

## 5. Curcumin Bioavailability

### 5.1. Animal Model Pharmacokinetics

**Figure 3:**
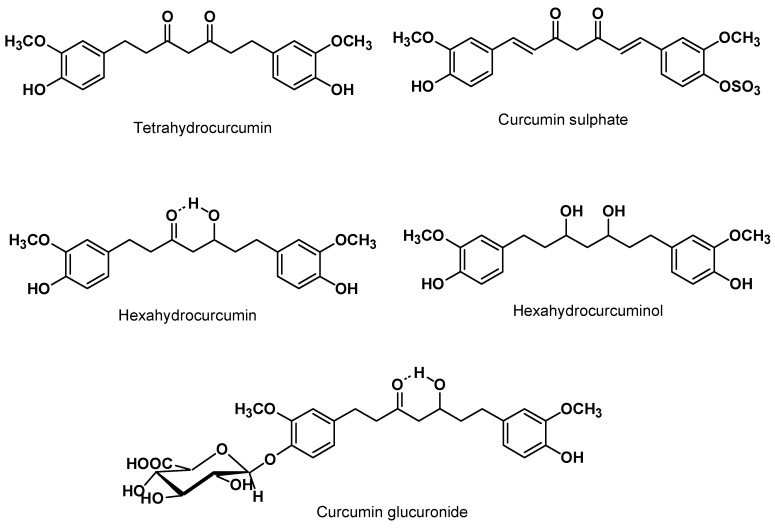
Structures of some important curcumin metabolites.

The absorption, distribution, metabolism and excretion studies of curcumin in recent years suggest that curcumin undergoes a rapid metabolism which has been suggested as the root cause of low bioavailability in systemic circulation. Curcumin administered orally at a dose of 1,000 mg/kg to rats resulted in approx. 75% of the dose being excreted in faeces and negligible amounts were detected in the urine [38]. In another study, absorption of oral curcumin reported on 60% and glucuronide and sulphate conjugates detected in the urine [39]. Ravindranath *et al.* reported that the majority of the oral dose was found to be excreted in faeces, while approx. 35% was excreted unchanged, and the remaining 65% excreted as metabolites of curcumin [40]. After intravenous and intraperitoneal administration of curcumin in rats, large quantities of this compound and its metabolites were excreted in the bile, mainly as tetrahydrocurcumin and hexahydrocurcumin glucuronides (Figure 3) [41,42]. These data offered evidence in favour of the hypotheses that curcumin undergoes transformation during absorption *via* the intestine and that it is possibly subject to enterohepatic recirculation [40]. These data are in accordance with the original hypothesis proposed by the researchers who studied the fate of curcumin in rats in 1978 [42].

Pan *et al.* have studied the intraperitoneal administration of curcumin (100 mg/kg) in mice, and have suggested that curcumin is first biotransformed to dihydrocurcumin and tetrahydrocurcumin, and that these compounds are subsequently converted to monoglucuronide conjugates [43]. Ireson *et al.* showed that small quantities of curcumin, hexahydrocurcumin, hexahydrocurcuminol, and hexahydro-curcumin glucuronide were present in plasma with higher levels of curcumin glucuronide and curcumin sulphate after oral dosing of curcumin in rats [44]. Interestingly, the transformation of curcumin to its metabolites occurred more extensively in rat hepatocytes grown *ex vivo* than in human hepatocytes. The same investigators extended their work by using suspensions of isolated human liver or gut microsomes and the results suggested that the metabolic reduction occurred very rapidly within minutes [45]. A study on a high dose of oral curcumin (2% in the diet, or approx. 1,200 mg/kg-BW) in F344 rats for 14 days showed that low nanomolar levels of parent compound are detectable in plasma, with concentrations in liver and colon mucosal tissue ranging from 0.1 to 1.8 nmol/g tissue [24].

Due to its poor systemic bioavailability after oral administration, many research groups have focused on ways to improve its bioavailability. Co-administration of oral curcumin with piperine, an alkaloid found in black pepper (*Piper nigrum*) and long pepper *(Piper longa)*, appeared to increase serum concentrations of curcumin in rodents. In a study using high doses of oral curcumin (2,000 mg/kg) in rats, the investigators found that co-administration of piperine increased systemic bioavailability by as much as 154% [46]. The mechanism of this effect has not been elucidated yet.

In summary, the systemic bioavailability of curcumin after oral dosing in rodents is low. Curcumin may undergo intestinal metabolism, and it appears it undergoes very rapid first-pass metabolism and excretion in bile. Co-administration with other agents or the use of different delivery systems may increase systemic bioavailability of curcumin.

### 5.2. Clinical Pharmacokinetics

In contrast to the extensive preclinical evidences presented, fewer pharmacokinetic data are available from human studies. In one of the studies, 2,000 mg of pure curcumin powder was administered to fasting volunteers and low curcumin concentrations in plasma (<10 ng/mL) were observed 1 h after the dose [46]. In the same study, concomitant administration of 20 mg of piperine appeared to increase bioavailability of curcumin in humans by 2,000%. In a study with higher doses of oral curcumin, Cheng *et al*. in Taiwan administered up to 8,000 mg of curcumin daily for three months to patients with preinvasive malignant or high-risk premalignant conditions. It was found that peak serum curcumin concentrations were achieved 1–2 h after oral intake as 1.75 μM and that levels gradually declined within 12 h [28]. In a dose-escalation study performed in the US, too low serum concentration of curcumin was observed (in some cases could not be detected) on administering 50–200 mg of oral micronized curcumin together with orange juice in healthy volunteers [47].

Two clinical phase I dose-escalation studies were conducted in patients with advanced colorectal cancer in Leicester, England. In the pilot study of 15 patients, standardized oral *Curcuma* extract (doses up to 180 mg of curcumin) was administered in a formulation that also contained volatile oils derived from *Curcuma* spp., daily for up to four months. No evidence of clinical toxicity attributable to the extract was detected, however, no evidence of detectable systemic bioavailability was observed either [48]. In a subsequent phase I study in 15 patients, a curcuminoid formulation was administered orally for up to four months, allowing rapid dose escalation and equating to curcumin doses between 450 and 3,600 mg daily [46]. Oral consumption of the highest dose of curcumin (3,600 mg daily) resulted in detectable levels of drug and conjugates in plasma, just above the limit of detection of the assay (approx. 0.63 ng/mL). Surprisingly, analysis of urine from patients consuming the same dose suggested the presence of curcumin and its conjugates in samples from patients receiving the 3,600 mg curcumin/day. Lower doses of curcumin resulted in no detectable urinary levels of the drug. In the six patients consuming 3,600 mg of curcumin daily, urinary levels varied between 0.1 and 1.3 μM (curcumin), 19 and 45 nM (curcumin sulphate), and 210 and 510 nM (curcumin glucuronide).

To determine levels of curcumin in gastrointestinal tissues, further studies were performed in patients undergoing operations for colorectal cancer who offered voluntary consent to have their tissues used for research purposes [49,50]. Twelve patients with histologically confirmed colorectal cancer received oral curcumin (450, 1,800, or 3,600 mg daily) for seven days before surgery. Levels of agent-derived species were determined in the peripheral circulation and in colorectal tissue obtained at the time of surgical resection. The concentrations of curcumin in normal and malignant colorectal tissue of patients consuming 3.600 mg daily of curcumin were 12.7 and 7.7 nmol/g tissue, respectively. Curcumin sulphate and curcumin glucuronide were identified in the intestinal tissues of these patients. Trace levels of curcumin were found in the peripheral circulation. In accordance with the data from preclinical models discussed earlier, these clinical results in both volunteers and patients suggest that curcumin has low systemic bioavailability in humans and that a dose of 3.600 mg curcumin per day achieved measurable levels of the parent compound in colorectal tissue. The same investigators examined the levels of curcumin in hepatic tissue after oral dosing in 12 patients with liver metastases from colorectal cancer who received 450–3.600 mg of oral curcumin daily for seven days before hepatic surgery [49]. They measured the levels of curcumin and its metabolites in portal and peripheral blood, bile, and liver tissue. Low nanomolar levels of curcumin and its glucuronide and sulphate conjugates were found in peripheral blood samples taken 1 h after seventh dose of curcumin and in portal blood samples taken 6–7 h after seventh dose of curcumin. In resected liver tissue, no parent drug was detected, but trace levels of metabolic products were found. This pilot study showed that the doses of oral curcumin required to produce hepatic levels sufficient to exert pharmacologic activity are probably not feasible in humans with this pharmaceutical formulation.

In summary, the results from several pilot and phase I clinical studies in volunteers and patients are consistent with the findings obtained with curcumin in preclinical models presented earlier. Collectively, they confirm that low systemic bioavailability is achieved after oral dosing, probably due to rapid first-pass metabolism and some degree of intestinal pre-metabolism. A daily oral dose of 3,600 mg of curcumin has been shown to result in detectable levels of curcumin in colorectal tissue and urine.

## 6. Curcumin in Inflammation and Cancer

### 6.1. Inflammation and Cancer

**Figure 4:**
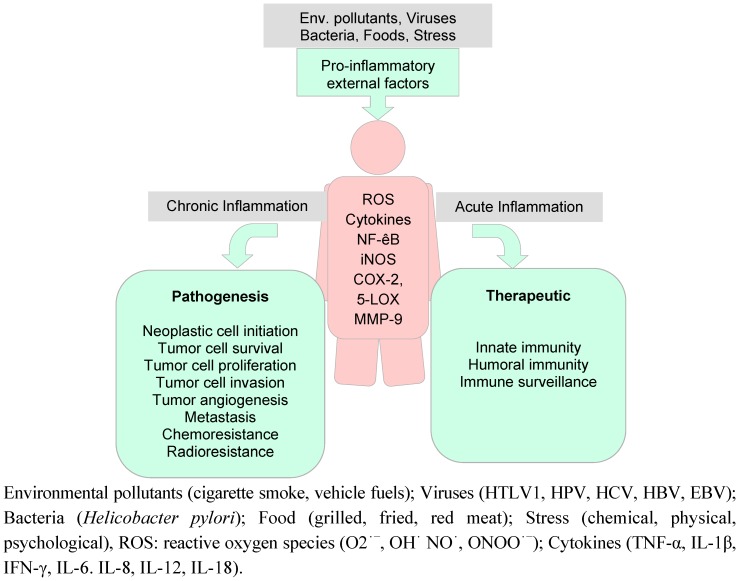
Chart showing external and internal pro-inflammatory factors and some of their biological responses.

The functional relation between inflammation and cancer was hypothesized by Virchow already in 1863 [51]. Recent data have expanded the concept that inflammation is a critical component of tumor progression. Many cancers arise from the site of inflammation, chronic irritation and infection. It should be noted that inflammation process is the essential part of the body physiology since acute inflammation is needed for the prevention from pathogens. In contrast to the acute inflammation, chronic inflammation is a low level inflammation that can persist over 20 to 30 years; eventually leading to cancer as well as other chronic diseases. Pro-inflammatory factors might be external such as environmental pollutants, viruses, bacteria, food, stress, *etc*. [52]. Several immune cells and their products guide and connect the inflammation reaction to the cancer progression. The principal target molecules of internal pro-inflammatory factors responsible for mediating the inflammation are the free radicals, IL-1β, TNF-α, NF-κB, and NSAID-activated gene-1 (NAG-1) [53,54,55]. Although these molecules are essential for normal cell regulation processes, uncontrolled and too high exposure to such molecules can lead to chronic diseases (Figure 4) [52].

Anti-oxidant enzymes such as superoxide dismutase (SOD), catalase (CAT) and glutathione peroxidase (GPX), together with the nonenzymatic system such as glutathione and vitamins (A, C, and E) defense against over-reaction of free radicals [56]. In addition to this, other molecules like polyphenols, ubiquinol-10, glucose and albumin, as well as minerals, such as selenium and zinc, can also counteract free radical activity.

Oxidative stress and oxidative damage caused by reactive oxygen species (ROS) are involved in the pathophysiology of many chronic inflammatory and degenerative disorders. The responsible free radicals are mainly ROS, such as superoxide anion (O_2_ֹ^−^), hydroxyl (OHֹ) and nitric oxide (NO**ֹ**) radicals along with non-free radical species such as hydrogen peroxide (H_2_O_2_) and nitrous acid (HNO_2_) [57]. The generation of ROS (particularly such as O_2_ֹ^−^, OHֹ NO**ֹ**, ONOOֹ^−^**)**, lipid peroxidation of cellular membranes, altered balance of anti-oxidant enzymes, such as an increase in cellular glutathione levels (GSH) and stress-induced activation of activator protein-1 (AP-1), play pivotal roles in the development of pathophysiology and degenerative disorders [58]. Another modulator, NO, a short-lived, lipophilic molecule, generated from L-arginine by various NADPH-dependent enzymes called NO synthases (NOSs) plays vital role in inducing cascades of pro-inflammatory molecules together with the physiologic functions such as vasodilatation, inhibition of platelet aggregation, neurotransmission, immune defence, and intracellular signalling [59]. NO is classified as a free radical species and some of its intermediates can damage DNA directly or interfere with DNA repair *via* protein damage [60,61]. Whereas high doses of NO (millimolar) seem to be cytotoxic and induce apoptosis, lower doses of NO (micro molar) can protect malignant cells from apoptosis *in vitro* through upregulation of COX-2 [62,63,64]. In addition, endothelial heme oxygenase-1 (HO-1) induced by cellular stress plays a critical role in defending the body against oxidant-induced injury during inflammatory processes [65].

These insights are fostering a new anti-inflammatory therapeutic approach to the cancer treatment. By the use of aspirin, a non-steroidal anti-inflammatory drug (NSAIDs), colon cancer risk can be reduced by 40%–50% by the COX-2 and COX-2 inhibition mechanism [66,67,68]. Another NASID, flurbiprofen, showed efficacy in inhibiting colon carcinogenesis due to its strong anti-metastatic effect together with the COX enzymes inhibition. Besides NSAIDs, the inhibitors of TNF-α, a highly studied pro-inflammatory cytokines and immune cells modulators, have also been targeted as the way of treatment for various cancers in the current clinical trails [69]. Therefore, anti-inflammatory drugs might open additional therapeutic option for the treatment of cancers and other chronic diseases.

In this context, in the last two decades, curcumin has been extensively studied as a potent anti-oxidant due to its ability to scavenge directly the ROS (such as O_2_ֹ^−^, OHֹ, NO**ֹ** and ONOOֹ^−^ radicals) and inflammatory agent by down regulating pro-inflammatory cytokines and transcription factors. Additionally, data have provided interesting insight into the immunomodulatory potential of curcumin. It can modulate the activation of T-cells, B-cells, macrophages, neutrophils, natural killer (NK) cells, and dendritic cells. Nevertheless, curcumin, at low doses, can also enhance antibody responses. These molecular evidences suggest that its reported favourable effects in cancers might be due to direct anti-oxidative and anti-inflammatory effects, as well in part to its ability to modulate the immune systems.

### 6.2. Preclinical Anti-Oxidant and Anti-Inflammatory Activities on Curcumin

#### 6.2.1. Anti-oxidant activity

Oxidative stress and oxidative damage are involved in the pathophysiology of many chronic inflammatory and degenerative disorders, particularly such as cancer. The generation of ROS, particularly O_2_^−^ and OHֹ, play important roles in the development of cancer [54,55]. Therefore in order to prevent from the oxidative damage of DNA, lipid or protein the effects of these free radicals can be diluted by the anti-oxidant mechanism.

Curcumin at the concentration of 10 μM inhibited ROS generation in rat peritoneal macrophages [70]. Similar effects have been observed in red blood cells [71]. Curcumin has also been shown to scavenge O_2_^−^ and OHֹ radicals [72,73]. In contrary, a few reports showing curcumin as pro-oxidant indicated that the pro-oxidant and anti-oxidant effects of curcumin are dependent on dose and the chemical environment (e.g., availability of free Cu^2+^ ions) [74,75]. Another free radical, NO, also plays important roles as an oxidant, inflammatory- and immune-modulator. Preclinical studies have suggested that curcumin may inhibit induction of macrophage NOS activity at concentrations of 1–20 μM [76]. In mice, oral administration of an aqueous alkaline solution of curcumin, notably at a tiny dose of 92 ng/g-BW strongly inhibited murine hepatic lipopolysaccharide-induced *iNOS* gene expression [58]. As inhibition of *i*NOS activity may represnt a mechanism of intervention during carcinogenesis, apparent activity of curcumin at low concentrations would have considerable implications for cancer chemoprevention.

Endothelial heme oxygenase-1 (HO-1) is a protein induced by cellular stress. Its main action is the degradation of heme to the anti-oxidant biliberdin and the vasoactive molecule carbon monoxide (CO) [7]. Curcumin showed a dose- and time-dependent increase in *HO-1* mRNA, protein expression, and heme oxygenase enzymatic activity in bovine aortic endothelial cells and human proximal renal tubular cells at the concentration of 1–8 μM through the NF-κB pathways and transcriptional mechanisms [77]. Increased heme oxygenase activity also appears to play an important role in curcumin-mediated cytoprotection against oxidative stress and NO-induced toxicity or apoptosis [78].

Interestingly, although curcumin shows anti-oxidant properties by providing hydrogen radicals and scavenging free radicals, there are a growing number of evidences that it can act as pro-oxidant under certain condition by generating ROS to show anticancer activity [79,80,81,82]. The balance between anti-oxidant and pro-oxidant activity must be considered when planning intervention trials in healthy volunteers, particularly if pro-oxidant activity results in potentially damaging effects, as shown in biomarkers such as oxidative DNA adduct levels [83].

#### 6.2.2. Anti-inflammatory effects by inhibition of arachidonic acid pathways

**Figure 5:**
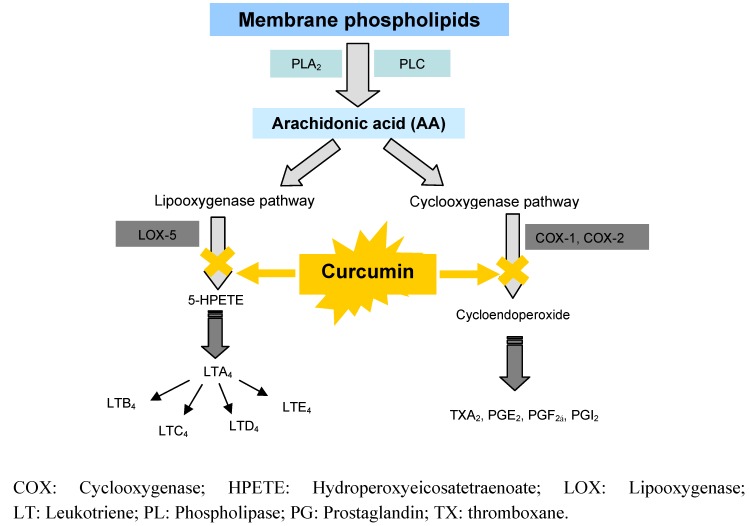
Flow diagram showing inhibitory effect of curcumin on arachidonic pathways.

The arachidonic acid metabolism consists of two well-described pathways, the cyclooxygenase (COX) and the lipooxygenase (LOX) pathways. Cyclooxygenase is the key enzyme involved in the COX pathway, converting arachidonic acid to prostaglandins and thromboxanes (Figure 5). There are two COX isozymes, namely COX-1 and COX-2. The COX-1 is a constitutive isoform expressed in most tissues; its inhibition results in adverse effects such as gastrointestinal ulcers or impairment of renal blood flow. Conversely, COX-2 is inducible at sites of inflammation by cytokines and intracellular signals; it can also be induced in various normal tissues by the hormones of ovulation and pregnancy, growth factors, oncogenes, and tumour promoters [84]. COX-2 is constitutively expressed only in brain and spinal cord tissue. COX-2 over expression has been implicated in the carcinogenesis of many tumours such as in colon, rectum, breast, head and neck, lung, pancreas, stomach, and prostate [85].

It has been shown that curcumin is able to inhibit induction of COX-2 gene expression in oral and colon epithelial cells [86,87]. At a concentration of 20 μM, curcumin showed a strong inhibition of chemically induced PGE_2_ production in colon cells [40]. In a study in human colon carcinoma cell lines carried by Lev-Ari *et al*., incubation of HT29 cells and SW480 cells with different concentrations of curcumin, resulted in inhibition of PGE_2_ synthesis, down regulation of COX-2 protein levels, and increased apoptosis of those cells that constitutively express COX-2 protein [87]. One of the implicated mechanisms for COX-2 down regulation is inhibition of the activity of the I*κ*K signalling complex responsible for phosphorylation of I*κ*B and subsequently the activation of the transcription factor NF-κB [88]. This finding was also supported by the fact that commonly used anti-inflammatory drugs such as aspirin and salicylates, which inhibit the activity of IκB kinase-β, have also been linked to a decreased incidence of colorectal cancer [89].

Apart from the well known roles of COX-2 [84], later studies suggest that the COX-1 isozyme also plays role in inflammation and carcinogenesis; indeed the balance between the metabolic products of COX-1 and COX-2 catalysis appears important in physiologic function and response to inflammation. Curcumin and some of its analogues do appear to inhibit COX-1 transcription [88,90]. Such inhibition is important, as it has been linked to a potential influence on the local spread of malignancy and the communication between malignant cells and their neighbouring stromal cells [91,92].

Numerous studies have indicated that the transcription factor, NF-κB plays important role to induce inflammation and is constitutively active in patients with cancer [93]. The role of NF-κB in suppression of apoptosis, tumour growth, invasion, angiogenesis, and metastasis, *via* a variety of downstream effectors, is well documented [94,95,96,97]. Therefore, an agent that can target NF-κB is of interest for the treatment of pancreatic cancer. From a philosophical standpoint, as cancer and most chronic diseases have a multifactor aetiology, natural diet-derived agents such as curcumin that act at multiple cellular levels may stand a better chance of improving the prevention or management of these diseases than agents that affect a single cellular target. A pleiotropic activity in the cell is provided by ability of curcumin to inhibit multiple levels of the NF-κB, AP-1, and JNK signalling pathways [98,99,100].

### 6.3. Clinical Studies on Curcumin

#### 6.3.1. Anti-oxidant activity

In a randomized controlled trial conducted in the US by using two diet-derived polyphenolic agents, the renoprotective and anti-oxidant properties of curcumin, in combination with the natural bioflavonoid quercetin, were tested in dialysis-dependent patients with cadaveric renal transplants [101]. It is known that the implicated mechanisms in renal graft rejection include alloimmune (T-cell-mediated cytotoxicity) and non-immune factors (e.g., cytomegalovirus infection, endothelial injury, and progressive atherosclerosis as a result of oxidative stress) [102,103]. In this study, both early graft and delayed graft functions were significantly improved in the drug (480 mg of curcumin and 20 mg of quercetin) treated group comparing to placebo group [101]. Another interesting observation was that tremor and neurotoxicity appeared to be less common in the drug treated group than in the control group. The investigators suggested that HO-1 induction may play a role in the improved early outcomes of cadaveric renal recipients treated with curcumin and bioflavonoids [101,104].

The anti-oxidant properties of curcumin were demonstrated in another pilot study in patients with chronic, non-alcoholic, tropical pancreatitis. In this study, 20 patients were randomized to receive either 500 mg of oral curcumin in combination with 5 mg piperine or placebo for up to six weeks [105]. The researchers evaluated the erythrocyte levels of malondialdehyde (MDA) and glutathione (GSH) as well as effects on clinical pain patterns for assessment of abdominal pain. The MDA and GSH levels in the body are general indicators of lipid peroxidation and anti-oxidant potential, respectively. The results of this study showed that patients treated with curcumin and piperine had significantly lower levels of MDA compared with the placebo group. Conversely, erythrocyte GSH levels remained unaltered, analogous to studies that showed that high-dose curcumin did not result in glutathione-*S*-transferase (GST) levels reduction. In this study, pain severity was unaffected by curcumin consumption. No side effects were reported by the patients, nor laboratory toxicities documented on blood tests done before and after the treatment course [105].

Anti-oxidant effects of curcumin in preclinical studies are well documented; however a limited report appeared to explain that biomarker directly related to the anti-oxidative effects.

#### 6.3.2. Anti-inflammatory activity

Suppression of the inflammatory response by curcumin as discussed in the preclinical studies involves inhibition of the induction of COX-1, COX-2, *i*NOS, and production of cytokines such as interferon-*γ* [90,106]. It seems that many effects are achieved *via* suppression of the Janus kinase (JAK)-STAT signalling cascade *via* its effect on the Src homology 2 domain-containing protein tyrosine phosphatases (SHP)-2 [107]. In human multiple myeloma cells, curcumin has been shown to inhibit STAT3 phosphorylation and therefore to down regulate IL-6 production [44].

The effect of oral curcumin on inflammatory diseases has been investigated in a number of clinical studies in various patient groups. In a pilot open-label study conducted in New York, oral curcumin was given to five patients with ulcerative proctitis and five patients with Crohn disease (CD) [108]. All the patients with proctitis noticed an improvement, and 80% of patients with CD showed better inflammatory profiles [108]. Subsequently, a randomized double-blind, placebo-controlled study was performed in patients with ulcerative colitis (UC) [109]. Of 89 patients enrolled, half received 1,000 mg of oral curcumin twice a day in combination with sulfasalazine or mesalazine, two aminosalicylates that are standard treatments for UC, and the other half of the patients received an aminosalicylate plus placebo. The 6-month treatment schedule showed that patients in the curcumin group had a significantly reduced rate of relapse at six months (4.65% *vs*. 20.51% in the placebo group) [109].

In a study in postoperative patients, a significant anti-inflammatory effect was demonstrated in those receiving 400 mg of oral curcumin thrice daily for five days compared with placebo [110]. Using similar methods, considerable improvements in the patients’ symptoms were demonstrated in a double-blind study in India on patients with rheumatoid arthritis. In this study, 1,200 mg of curcumin was administered four times daily to 18 patients with rheumatoid arthritis for two weeks, resulting in symptomatic improvement without apparent toxicity [27]. The anti-inflammatory properties of curcumin were demonstrated in two further studies examining the effects of oral curcumin on ophthalmologic diseases. In one of those, 375 mg of curcumin was given orally thrice daily to patients with chronic anterior uveitis for three months, resulting in a suggestion of improvement in the condition with comparable efficacy to that of steroids, which are generally regarded as standard treatment [111]. In a subsequent study by the same team, the same dose of curcumin (375 mg) was administered to eight patients with idiopathic inflammatory orbital pseudotumors for 6–22 months [112]. Half of the patients showed complete responses up to two years of follow-up. It is known that in patients with active psoriasis, increased activity of the enzyme phosphorylase kinase (PhK) mediates and triggers molecular mechanisms for continuous cell migration and proliferation. In an early preclinical study, Reddy and Aggarwal demonstrated that curcumin is a selective inhibitor of PhK [113]. More recently, PhK activity was assessed in 40 patients who were divided into four groups: a group of active untreated psoriasis, one of resolving psoriasis treated with calcipotriol [vitamin D3 analogue], a group of patients receiving curcumin, and a control group with healthy subjects [114]. This study showed that PhK activity was much higher in untreated patients, lower in the groups treated with calcipotriol and curcumin, and even lower in the control group. The decrease in activity of PhK caused by curcumin and calcipotriol was associated with a suppression of keratinocyte transferrin receptor, the severity of parakeratosis, and the density of the epidermal cytotoxic CD8^+^ T cells, all considered clinical hallmarks of psoriatic activity [114].

In contrast to the systemic anti-oxidant properties of oral curcumin, it has been suggested that topical application of curcumin may result in increased formation of ROS in the skin when used in combination with other therapies [115]. Such potentially pro-oxidant effects may relate to dose or conditions, as discussed earlier. In a study performed in Bradford, England, 15 Asian patients with acute vitiligo consumed turmeric daily and were treated with topical application of low-dose UVB-activated pseudocatalase (PC-KUS) for six months [115]. None of these patients showed any significant improvement of vitiligo. Eight patients were advised to stop turmeric consumption and continue with topical PC-KUS only twice daily. This led to a clinical improvement within two months and to an almost complete repigmentation at six months in six of the eight patients, leading the authors to suggest that turmeric was having an antagonistic effect on the treatment of the disease by PC-KUS.

In forty five patients with peptic ulcer-like symptoms treated with 600 mg of oral curcumin, five times per day, ulcer-like symptom disappeared in 12 patients after four weeks of treatment. This response increased to 72% of patients after eight weeks of treatment, and up to 76% of patients at 12 weeks of treatment. The remaining 20 patients exhibited gastritis, erosions, or dyspepsia rather than definite ulcers as on the beginning of the treatment also reported symptomatic improvement within two weeks of the 4-week course of curcumin treatment. No treatment-related toxicities were noticed [116].

#### 6.3.3. Clinical studies: Anti-cancer effects

As it is evident that curcumin expresses anti-oxidant, anti-inflammatory, anti-antigenic, anti-mitotic and anti-metastatic activities *in vitro* and in animal experiments, curcumin thus might be a promising molecule for the prevention and treatment of cancer in humans. Kuttan *et al.* observed the reduced size of the lesions in 10 of the 62 patients receiving topical turmeric/curcumin in oral cancers and leukoplakia, however the report is lacking the control group and standard method of curcumin preparation [117].

A phase I clinical study performed in Taiwan investigated the potential anti-carcinogenesis activity of curcumin in patients with preinvasive malignant or high-risk premalignant conditions [28]. Twenty five patients with recently resected cancer of the bladder, Bowen disease of the skin, uterine cervical intraepithelial neoplasia, intestinal metaplasia of the stomach, or oral leukoplakia, were administered curcumin in doses of 1,000 to 8,000 mg (500 mg of synthetic curcumin per capsule, 99% purity) daily for three months. Although no toxicities were reported for doses up to 8,000 mg per day, higher daily doses were not acceptable to patients on account of the sheer number of capsules needed. Histologic improvement of the premalignant lesions was noted in one of two patients with presumed bladder carcinoma *in situ*, two of seven patients with oral leukoplakia, one of six patients with stomach intestinal metaplasia, one of four patients with cervical intraepithelial neoplasia (CIN), and two of six patients with Bowen disease of the skin. On the contrary, one of four patients with CIN, and one of seven patients with oral leukoplakia developed malignancy despite the treatment. Limitations for drawing definite conclusions from this study are the small numbers of patients with each high-risk conditions studied and the possible bias from the interpreting pathologists, as the study was not blinded. Nevertheless, the results suggested biologic activity in the studied diseases. Further evidence to support the hypothesis that curcumin has activity against preneoplastic lesions is provided by a recent study performed at The Cleveland Clinic in Florida in patients with familial adenomatous polyposis (FAP) [118]. FAP is an autosomal dominant condition characterized by the development of numerous bowel adenomas that can transform to adenocarcinoma. Curcumin (480 mg), in combination with quercetin (20 mg), was administered three times per day to five patients with FAP. Four patients had the rectum preserved, and one had an ileoanal pouch. All patients showed a decrease in the number and the size of polyps compared with baseline figures [118]. These preliminary data strongly support the case for designing a randomized controlled trial of curcumin *versus* standard therapy for patients with FAP.

In a Phase II clinical study report of Dhillon at el., twenty-five patients each treated with 8000 mg per day curcumin orally for two months observed peaked plasma curcumin level from 22 to 41 ng/mL [119]. On continuing up to 18 months, two patients showed clinical biological activity. One had ongoing stable disease for more than 18 months; interestingly, one additional patient had a brief, but marked, tumour regression (73%) accompanied by significant increases (4- to 35-fold) in serum cytokine levels( IL-6, IL-8, IL-10, and IL-1 receptor antagonists). Curcumin down-regulated the expression of NF-κB, COX-2, phosphorylated signal transducer and activator of transcription 3 in peripheral blood mononuclear cells from patients. In conclusion, although this molecule is poorly absorbed, with low nanogram levels of circulating curcumin detected at steady-state, biological activity is evident. Collectively, these clinical trials highlight the level of current translational interest in studying the biologic potential of curcumin in the treatment of premalignant conditions and established malignancies.

In the review of Schehzad *et al.*, a list of ongoing Phase III clinical trials with curcumin against cervical, oral, pancreatic and colon cancers in India and Israel is reported, however, the results of these clinical trials have not been published at the time of this manuscript preparation [120].

The current review is focused mainly on the effects of curcumin in inflammation and its link to cancer in general, therefore it provides limited information on the details of the molecular mechanism(s) of action. Recently, Goel *et al*. have discussed in details the effects of curcumin on various cancers in human [121] and Wilken *et al*. reviewed the mechanism of anti-cancer properties of curcumin focused on head and neck squamous cell carcinoma [122]. Both reviews provide additional information on molecular mechanism of anticancer effects on curcumin.

## 7. Recent Advancements on Curcumin Formulations and Delivery Systems

Like many other natural polyphenols, curcumin is also poorly soluble in water. It is well established fact that the main limitation of broader use of curcumin-based formulations is its poor solubility and fast metabolism [123]. Therefore, in order to increase its solubility, stability and pharmacological activities, further research on improved formulations and delivery systems is needed to achieve its optimum therapeutic effects. In this section some recent progress in curcumin formulations and delivery system development is discussed.

In search for enhanced bioavailability of curcumin, the first step would be to improve its solubility. For that purpose, various classical techniques based on physical parameters such as heat, pH, and complexations with metal ions, polymers or serum have been applied to prepare more soluble curcumin formulations. In addition, chemical modifications of curcumin are carried out to prepare curcumin derivatives or analogues. Kurien *et al.* claimed that the solubility of curcumin and turmeric can be increased by 12-fold and 3-fold, respectively, by the use of heat without heat-mediated disintegration of curcumin [124]. For delivery of drugs *in vivo*, water is indisputably the simplest and the safest vehicle, therefore possibility of considering heat-solubilized curcumin for future *in vivo* and *in vitro* studies might be interesting [124]. Zebib *et al*. prepared complexes of curcumin with metal ions (Zn^2+^, Cu^2+^, Mg^2+^ and Se^2+^) that were found to be readily soluble in water-glycerol (1:1; w/w) and quite stable towards light and heat [125]. Complexes of curcumin with serum albumin significantly increased the solubility of curcumin and, at the same time, reduced the toxic effect of amphoterecin B by delaying the erythrocyte membrane damage [126]. Qiu *et al.* reported chemically modified 4-arylidene curcumin derivatives that were found to be more soluble and more potent anti-cancer targeted analogues [127]. In addition, several attempts have also been made to prepare chemically modified curcumin in order to increase its activity against cancer and NF-κB [128,129,130,131,132,133]. Safavy *et al.* developed water-soluble curcumin conjugates with two differently sized poly(ethyleneglycol) molecules. The soluble conjugates exhibited enhanced cytotoxicity as compared to curcumin alone and showed potentials in anti-cancer treatment [134].

Various novel delivery systems were proposed in recent years as means to improve bioavailability of curcumin. This review focuses on the systems aiming at improved anti-inflammatory and anti-cancer properties of curcumin.

Based on the material used in the methods of preparation, those delivery systems can be broadly classified as follows:

i) *Polymeric implantable delivery systems for curcumin*: Bansal *et al.* prepared curcumin in poly-(*ε*-caprolactone)-based implants aimed at subcutaneous grafting and evaluated the implants in rats. The maximum concentration of curcumin in liver was detected on day 4 post-implantation and the plateau was observed after seven days. The study confirmed the potential of polymeric implants to avoid oral route and provide sustained release of incorporated curcumin [123].
ii) *Micelles*: injectable curcumin-loaded poly(ethyleneoxide)-*b*-poly(*ε*-caprolactone) micelles for controlled delivery of curcumin prepared by Ma *et al.* confirmed that micelle-encapsulated curcumin retained its cytotoxicity in both mouse melanoma and human mantle cell lymphoma cell lines [135]. Curcumin-loaded poly(D,L-lactide-co-glycolide)-b-poly(ethylene glycol)-b-poly(DL-lactide-co-glycolide; PLGA-PEG-PLGA) micelles, developed by Song *et al.* showed improved area under the curve (AUC) and t_1/2_ *in vivo*. Moreover, micelles decreased curcumin uptake by liver and spleen, and at the same time, enhanced distribution of curcumin in lung and brain [136].
iii) *Nano-delivery systems*: Nanotechnology and nanomedicine offer potentials for development of nano sized delivery systems for curcumin. Shaikh *et al.* developed nanoparticles encapsulating curcumin prepared by the emulsion technique. The *in vivo* pharmacokinetics revealed that nanoparticles-incorportaed curcumin achieved a 9-fold increase in oral bioavailability as compared to curcumin administered with piperine as absorption enhancer [137]. Tsai *et al.* prepared an optimized polylactic-co-glycolic acid (PLGA) nano-formulation of curcumin which resulted in a 22-fold higher oral bioavailability in rats as compared to conventional curcumin [138]. Curcumin loaded dextran sulphate-chitosan nanoparticles showed preferential killing of cancer cells compared to normal cells, indicating potential in targeting [139].
  A very promising delivery system appears to be “nanocurcumin”, polymeric nanoparticle-encapsulated curcumin, readily dispersed in aqueous media and with confirmed anti-cancer potentials in preclinical *in vivo* models. Nanocurcumin retained the mechanistic specificity of free curcumin, inhibiting the activation of the seminal transcription factor NF-*κ*B and reducing steady state levels of pro-inflammatory cytokines like ILs and TNF-α [140]. Nanocurcumin developed by Bhawana *et al.* and prepared by wet-milling technique, in size range of 2–40 nm was shown to express stronger antimicrobial potential. It remains to be seen whether the same nanocurcumin will display enhanced anti-cancer activity as well [141]. A similar approach in reducing the size of curcumin crystals was proposed by Gao *et al.* to produce nanosupsensions for intravenous delivery [142].
  Wu *et al.* developed water-dispersible hybrid nanogels for intracellular delivery of curcumin aiming at photodermal therapy [143]. The hybrid combines optical label (Au/Ag bimetallic nanoparticle, polystyrene gel layer, polyethylene gel and provides potent cytotoxicity against B16F10 cells by combined chemo-phototermal treatment. Anti-inflammatory activity of curcumin was also found to be enhanced through delivery via o/w nanoemulsions, as evaluated in mouse ear inflammation model. In comparison, Tween-based formulations failed to achieve the same effect [144].
iv) Although *phospholipid-based delivery systems*, based on their size, mostly fall in the category of nanomedicine, due to the specificity of the carrier material, phospholipid vesicles are treated here a separate category. Several research groups have proposed curcumin-phospholipid complexes as means to improve curcumin delivery. Complexation of curcumin with phosphatidylcholine resulted in enhanced bioavailability, improved pharmacokinetics and increased hepatoprotective activity as compared to physical mixtures of curcumin and phosphatidylcholine [145]. Curcumin formulated with Meriva^®^ (phosphatidylcholine) showed increased bioavailability in rats [146]. Curcumin-phospholipid complex administered orally resulted in higher serum concentrations of curcumin as compared to uncomplexed curcumin. Moreover, the complex maintained the effective concentrations of curcumin over longer period of time [147]. However, the content of curcumin in complexes was found to be limited to about 17 and 32% (w/w), respectively, which is much lower than what could be achieved by liposomal encapsulation of curcumin.
  Phospholipid vesicles and lipid-nanospheres embedding curcumin improved intravenous delivery of curcumin to tissue macrophages, especially bone marrow and spleen macrophages [148]. Solid lipid nanoparticles (SLN) were also proposed as mean to enhance oral bioavailability of curcumin. Pharmacokinetic profile of curcumin on SLN in rats showed significant improvement as compared to solubilized curcumin [149]. In order to further enhance anti-cancer potential of curcumin, transferrin-mediated SLN containing curcumin were developed and their superiority confirmed in breast cancer cells [150]. Solid lipid nanoparticles were proposed for topical application of curcumin as well [151]. Solid lipid nanoparticles developed by Yadav *et al.*, were evaluated in rat model of inflammatory bowel disease and showed enhanced anti-angiogenic and anti-inflammatory activities [152].
  Probably one of the most studied delivery systems for curcumin delivery is liposomes. Liposomes are well established delivery system able to incorporate poorly soluble drugs and enable their aqueous medium-based administration [153]. Curcumin is expected to accommodate itself inside the hydrophobic interior of liposomes, resulting in higher drug loading capacity [154]. Liposomal curcumin was also reported to have higher stability than free curcumin in phosphate buffer saline, human blood, plasma and RPMI-1640 medium with 10% calf serum [155]. Li *et al.* developed liposomal delivery system for curcumin aiming at intravenous administration. Liposomal curcumin consistently suppressed NF-κB binding and decreased the expression of NF-*κ*B-regulated gene products, including COX-2 and IL-8, both of which have been implicated in tumour growth/invasiveness. The activity of liposomal curcumin was equal to or better than that of free curcumin at equimolar concentration. Antitumor and anti-angiogenesis effects were suppressed *in vivo* and based on their results the authors propose that liposomal curcumin for systemic delivery provides the rationale for the treatment of patients suffering from pancreatic carcinoma [156]. Li *et al.* developed liposomal curcumin which showed dose-dependent growth inhibition and apoptosis in the two human colorectal cancer cell lines (LoVo and Colo205 cells) and synergetic effect with oxaliplatin, a standard chemotherapy for the malignancy. In *in vivo* study, liposomal curcumin significantly inhibited tumour growth in Colo205 and LoVo xenografts in mice [157]. Thangapazham *et al.* developed liposomal delivery system for curcumin in which liposomes were coated with prostate membrane specific antigen specific antibodies to achieve targeting. The superiority of such a system was evaluated in two human prostate cancer cell lines. Antibody-coated liposomes showed 10-fold more anti-proliferative activity in human prostate cancer cell lines (LNCaP and C4-2B) compared to non-liposomal curcumin. It was also observed that LNCaP cells were relatively more sensitive to liposomal curcumin than C4-2B cells [158]. Wang *et al.* reported on liposomal formulation of curcumin able to suppress the growth of head and neck squamous cell carcinoma (HNSCC) in *in vitro* study in dose-dependent manner and also able to suppress the activation of NF-κB without affecting the expression of pAKT. The expression of cyclin D1, COX-2, MMP-9, Bcl-2, Bcl-xL, Mcl-1L and Mcl-1S were reduced. Nude mice xenograft tumours were suppressed after 3.5 weeks of treatment with *i.v.* liposomal curcumin, and no demonstrable toxicity of liposomal curcumin was detected. The authors speculated that liposomal curcumin is a viable non-toxic therapeutic agent for HNSCC [159]. Takahashi *et al.* developed liposomal delivery system for oral administration of curcumin, incorporating up to 68% of curcumin. Faster rate and better absorption after oral administration in rats where achieved as compared to non-liposomal curcumin. These results indicated that liposomal encapsulation enhanced the gastrointestinal absorption of curcumin [160]. A liposome-based intravenous formulation of bis-demethoxy curcumin analogue showed better hepatoprotective activity comparing to its free form [161]. Narayanan *et al.* proposed interesting approach for the treatment of prostatic adenocarcinoma. Combination of liposomal forms of curcumin and resveratrol significantly decreased prostatic adenocarcinoma *in vivo*. *In vitro* studies revealed that curcumin in combination with resveratrol effectively inhibited cell growth and induced apoptosis. These findings suggested that liposomal phytochemicals-in-combination may reduce prostate cancer incidence [162]. Mourtas *et al.* proposed novel curcumin-decorated nanoliposomes with very high affinity for amyloid-β-42 peptide as vectors for targeted delivery of Alzheimer disease treatment. This approach opens the possibility to further explore the potential of vesicle-surface available curcumin in various cancer treatments [163].

Based on the above listed studies, it is evident that novel delivery systems enhance stability, bioavailability and cellular uptake of curcumin. However, in spite of all these promising results, novel delivery systems for curcumin has not yet reached clinical evaluation. We would also like to point out that dozens of patents on lipid-based formulations were granted in recent years, but those formulations are not discussed in this review.

## 8. Conclusions

The proposed hallmarks of cancer comprise six biological capabilities acquired during multistep development of human tumours. They include sustaining proliferative signalling, evading growth suppressors, resisting cell death, enabling replicate mortality, inducing angiogenesis and activating metastasis. Genome instability and immune guided inflammatory reactions lead to the development of tumour microenvironment [164]. In the last three decades, the introduction of mechanism-based targeted therapies to treat human cancers provided better understanding of the mechanism in cancer pathogenesis. Some examples of selected therapeutic targeting are listed below (Table 1).

**Table 1:** The selected therapeutic targets designed for the cancer treatment.

Therapeutic targeting	Effects on
Selective anti-inflammatory drugs	Tumour promoting inflammation
Telomerase inhibitors	Enabling replicate immortality
Inhibitors of HGF/c-Met	Activating invasion and metastasis
Inhibitors of VEGF signalling	Inducing anti-angiogenesis
Inhibitors of PARP	Genome instability and mutation
Proapoptic BH3 mimetics	Resisting cell death
Aerobic glycolysis inhibitors	Deregulating cellular energetics
EGFR inhibitors	Sustaining proliferative signaling
Cyclin-dependent kinase inhibitors	Evading growth suppressor
Immune activating anti-CTLA4 mAb	Avoiding immune destruction

This table is modified from the illustration given by Hanahan and Weinberg [164].

In response to the therapy, cancer cells may also reduce their original dependence on a particular capability, becoming more dependent on another hallmark; this representing a quite different form of acquired drug resistance. This concept is exemplified by recent discoveries [165,166,167]. Therefore, a multi-targeted molecule, such as curcumin, might be better choice for the treatment of cancer.

The long history of curcumin mass use, and recent clinical trials have shown its excellent safety index. Over the last few years, a number of studies have provided evidence for several pharmacological properties of curcumin including chemosensitizing, radiosensitizing, wound healing, antimicrobial, antiviral, antifungal, and anti-inflammatory activities [168]. Curcumin has been shown to inhibit several cell signalling pathways at multiple levels, affecting the expression and activity of cellular enzymes such as COX and GSTs, and influencing immunomodulation, angiogenesis, and cell-cell adhesion. Its ability to affect gene transcription and to induce apoptosis is of particular relevance to cancer chemoprevention and chemotherapy.

Recently, research on multicomponent drugs, especially phytochemical extracts or combination of targeted ingredients have provided more insight on additive and synergetic effects achieving optimum therapeutic effects. Use of a standardized extract such as Polyphenone E, a topical antiviral cream based on purified green tea extract has been approved as a prescription drug by FDA in 2006, would be the new trend in pharmaceutical sciences [169]. Similarly, potentially beneficial interactions between diet-derived polyphenols and other drugs and between individual components of the human diet have been identified. Examples of this concept include the combination of curcumin with the green tea extract, epigallocatechin-3-gallate (EGCG) [170]; curcumin combined with the flavonoid quercetin, found in apples, onions, and citrus fruits [118]. Interactions relevant to combinatorial treatment with curcumin might be important due to alteration in absorption, cellular and biological responses. For example, the combination of curcumin and genistein (a natural product derived from soya beans) appears to strongly inhibit growth of human breast MCF-7 cells synergistically [171]. It is also evident that the toxic effect of drugs like 5-flurouracil vinorelbine can be reduced by combined treatment with curcumin [172,173].

The experience in cancer research revealed that targeted treatment of cancer could not often be achieved due to the adaptation and modification by the cancer cells during treatment. In this connection, curcumin, a pleiotropic molecule, seems quite promising. In addition, its additive and synergistic properties with other phytochemicals or routinely used toxic drugs explored in recent preclinical test are creating a new philosophy of safe and effective treatments. Therefore, the choice of curcumin as chemopreventive and potentially anti-cancer molecule seems promising. However, its formulation and delivery systems needed to be further improved in preclinical and clinical studies in order to make this curry spice into an anti-cancer drug.
